# Synthesis and Luminescence Properties of Eu^2+^-Doped Sr_3_MgSi_2_O_8_ Blue Light-Emitting Phosphor for Application in Near-Ultraviolet Excitable White Light-Emitting Diodes

**DOI:** 10.3390/nano12152706

**Published:** 2022-08-06

**Authors:** Chou-Yuan Lee, Chia-Ching Wu, Hsin-Hua Li, Cheng-Fu Yang

**Affiliations:** 1 School of Big Data, Fuzhou University of International Studies and Trade, Fuzhou 350202, China; 2 Department of Applied Science, National Taitung University, Taitung 95092, Taiwan; 3 Department of Chemical and Materials Engineering, National University of Kaohsiung, Kaohsiung 811726, Taiwan; 4 Department of Aeronautical Engineering, Chaoyang University of Technology, Taichung 413310, Taiwan

**Keywords:** Sr_3_MgSi_2_O_8_, Eu_2_O_3_, phosphor, blue emission

## Abstract

In this study, [Sr_0.99_Eu_0.01_]_3_MgSi_2_O_8_ phosphors were sintered at 1200–1400 °C for 1–5 h by using the solid-state reaction method. The crystallinity and morphology of these phosphors were characterized through X-ray diffraction analysis and field-emission scanning electron microscopy, respectively, to determine their luminescence. The photoluminescence properties, including the excitation and emission properties, of the prepared phosphors were investigated through fluorescence spectrophotometry. The α-Sr_2_SiO_4_, Sr_2_MgSi_2_O_7_, and Sr_3_MgSi_2_O_8_ phases coexisted in the [Sr_0.99_Eu_0.01_]_3_MgSi_2_O_8_ phosphors, which were synthesized at low temperatures. The particles of these phosphors had many fine hairs on their surface and resembled *Clavularia viridis*, which is a coral species. Transmission electron microscopy and energy dispersive X-ray spectroscopy indicated that the fine hairs contained the Sr_2_SiO_4_ and Sr_2_MgSi_2_O_7_ phases. However, when the [Sr_0.99_Eu_0.01_]_3_MgSi_2_O_8_ phosphors were sintered at 1400 °C, the Sr_3_MgSi_2_O_8_ phase was observed, and the Eu^2+^-doped Sr_3_MgSi_2_O_8_ phase dominated the only broad emission band, which had a central wavelength of 457 nm (blue light). The emission peaks at this wavelength were attributed to the 4f^6^5d^1^–4f^7^ transition at the Sr^2+^(I) site, where Sr^2+^ was substituted by Eu^2+^. The average decay time of the synthesized phosphors was calculated to be 1.197 ms. The aforementioned results indicate that [Sr_0.99_Eu_0.01_]_3_MgSi_2_O_8_ can be used as a blue-emitting phosphor in ultraviolet-excited white light-emitting diodes.

## 1. Introduction

White light-emitting diodes (W-LEDs) have replaced conventional incandescent and fluorescent lamps for general illumination. Historically, artificial lighting is energy-intensive, with incandescent lamps exhibiting a luminous efficiency of only 2% and quartz halogen and fluorescent lamps reaching 4% and 15%, respectively, with most of the energy input converted to waste heat. In contrast, solid-state lighting based on W-LEDs currently attains ∼32% luminous efficiency. W-LEDs are a novel high-efficiency lighting system and fourth-generation illumination source with many advantages, including a long lifetime, high rendering index, high luminosity efficiency, low energy consumption, chemical stability, thermal stability, and eco-friendliness [1,2,3]. W-LEDs have superior luminescence characteristics relative to other lighting sources [4]. W-LEDs have many applications in various domains, such as lighting [5], biomedicine [6], communication [7], liquid crystal displays (as backlight sources) [8], and architecture [9]. However, there are several important luminescence parameters that characterize and determine the quality of W-LEDs, including luminous efficacy (LE), color rendering index (CRI), and correlated color temperature (CCT) [10,11].

Two main methods are currently used for producing W-LEDs. The first and most commonly adopted method involves producing W-LEDs by using a blue light-emitting diode chip and yellow light-emitting YAG: Ce^3+^ phosphor; however, the W-LEDs produced using this method have low CRI values (70 to 80) and a CCT value of 7750 K, because the light produced by them does not contain a red component [12,13]. The low CRI value of W-LEDs at a low color temperature limits their possible applications; however, many efforts have been made to overcome this disadvantage. W-LEDs produced using the second method of red (R), blue (B), and green (G) phosphors emit “warm” white light with a high CRI. Phosphor materials that can be effectively excited by ultraviolet or blue light to emit strong R, G, and B light have attracted considerable research attention [14,15,16].

M_3_MgSi_2_O_8_ (M = Ca, Sr, Ba) phosphors were first reported in 1957 [17]. Alkali earth silicates are crucial hosts for rare-earth-doped phosphors because of the inherent advantages of these silicates, such as excellent chemical and thermal stability as well as the low price of high-purity silicate [18]. Klasensetal investigated the photoluminescence (PL) properties of Pb^2+^-, Mn^2+^-, Tl^+^-, and Sb^3+^-activated M_3_MgSi_2_O_8_ (ternary silicates). In addition to the Pb^2+^-activated M_3_MgSi_2_O_8_, none of the other silicates could emit light efficiently. Moreover, Klasensetal found that a substantial amount of Ca^2+^ in Ca_3_MgSi_2_O_8_ can be replaced by Ba^2+^, whereas only a slight amount of Ba^2+^ in Ba_3_MgSi_2_O_8_ can be replaced by Ca^2+^ [17].

Europium oxide **(**Eu_2_O_3_) is a highly useful doping material. When added to host materials as an activator, Eu_2_O_3_ has different ionic states and causes synthesized phosphors to produce different emission colors. Most Eu_2_O_3_-doped materials synthesized in the atmosphere behave as Eu^3+^-activated phosphors and emit red [19,20,21] or near-infrared [22] radiation. When Eu_2_O_3_-doped materials are synthesized in a reducing atmosphere, Eu^3+^ ions are reduced to Eu^2+^ ions, which results in the formation of Eu^2+^-activated phosphors that emit blue light [23,24] or green light [25]. Many Eu^2+^-activated materials and relevant synthesis methods have been developed to investigate highly efficient blue or green phosphors. A study that examined mixtures of Eu^2+^-activated Ba_3_MgSi_2_O_8_ and Ca_3_MgSi_2_O_8_ found that Ba_3_MgSi_2_O_8_ has a higher PL emission intensity and shorter peak emission wavelength (437 nm) than does Ca_3_MgSi_2_O_8_ (peak emission wavelength of 475 nm).

In the present study, we synthesized Eu_2_O_3_-doped Sr_3_MgSi_2_O_8_ phosphors by using the solid-state reaction method at high temperatures, and investigated the crystal structure and PL properties of these phosphors. The effects of the synthesis temperature and time on Eu_2_O_3_-doped Sr_3_MgSi_2_O_8_ phosphors were investigated. When Eu_2_O_3_-doped Sr_3_MgSi_2_O_8_ was synthesized in a reducing atmosphere, Eu^3+^ ions were reduced to Eu^2+^ ions, and the synthesized phosphors emitted strong blue light. [Sr_1−x_Eu_x_]_3_MgSi_2_O_8_ might be a promising blue phosphor for RGB-W-LEDs.

## 2. Experimental

### 2.1. Preparation of the [Sr_1−x_Eu_x_]_3_MgSi_2_O_8_ Phosphors

In this study, [Sr_0.99_Eu_0.01_]_3_MgSi_2_O_8_ phosphors were synthesized using the solid-state reaction method. The raw materials used in this synthesis were SrCO_3_ (Sigma-Aldrich, St. Louis, MO, USA, 99.99%), MgO (Sigma-Aldrich, USA, 99.99%), SiO_2_ (Sigma-Aldrich, USA, 99.99%), and Eu_2_O_3_ (Sigma-Aldrich, USA, 99.99%) powders. These powders were mixed and ground in deionized water for 1 h by using the ball-milling method. ZrO_2_ balls with a diameter of 5–8 mm were used to grind the powders. The powder mixture was then dried at 120 °C for 24 h in an oven. After drying, the mixture was ground in an agate mortar for 1 h and then calcined at 850 °C for 2 h. The mixture was placed in alumina crucibles and put in the tubular furnaces. Then, a vacuum was created in the tubular furnaces by using the mechanical pump. Finally, the reducing gas (4 vol% H_2_/96 vol% N_2_) was led into the tubular furnaces, and the mixture was sintered at 1200–1400 °C for 1–10 h in a reducing atmosphere.

### 2.2. Measurements

The crystalline structures of the prepared [Sr_0.99_Eu_0.01_]_3_MgSi_2_O_8_ phosphors were investigated using a ceramic X-ray diffraction (XRD) source that emitted CuKα radiation (*λ* = 1.5406 Å). The microstructures of the phosphors were analyzed through field-emission scanning electron microscopy (FE-SEM) and high-resolution transmission electron microscopy (HR-TEM). The PL spectra and PL excitation (PLE) spectra were obtained using a Hitachi F-7000 spectrofluorometer with a 150-W xenon lamp as the light source. The luminance and International Commission on Illumination [Commission Internationale de l’Eclairage (CIE)] coordinates were measured using the CS-100A Konica Minolta chroma meter. All the measurements were performed at room temperature.

## 3. Results and Discussion

**Figure 1:**
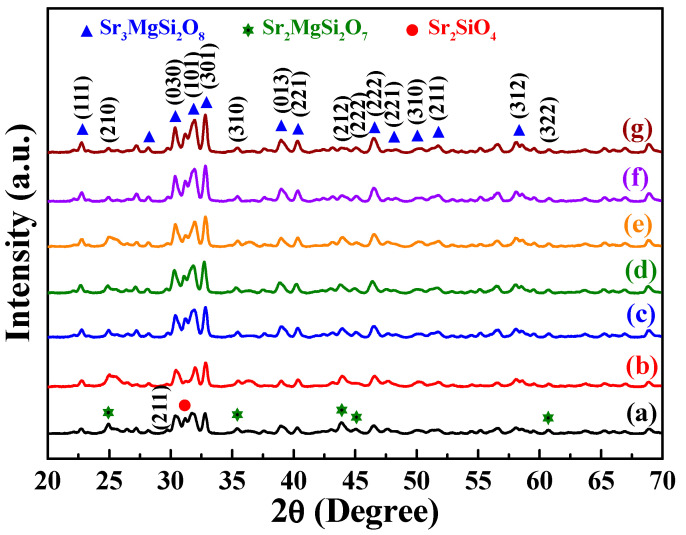
XRD patterns of the [Sr_0.99_Eu_0.01_]_3_MgSi_2_O_8_ phosphors sintered for different durations: (a) 1, (b) 2, (c) 3, (d) 4, (e) 5, (f) 6, and (g) 10 h.

**Figure 2:**
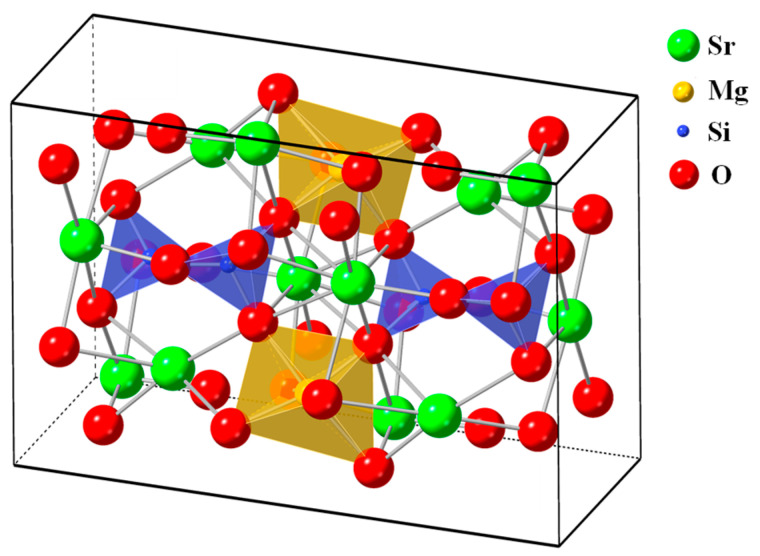
Crystal structure of the [Sr_0.99_Eu_0.01_]_3_MgSi_2_O_8_ phosphors.

The XRD patterns of the prepared [Sr_0.99_Eu_0.01_]_3_MgSi_2_O_8_ phosphors were obtained to verify their crystal structures. Figure 1 shows the diffraction peaks of the [Sr_0.99_Eu_0.01_]_3_MgSi_2_O_8_ phosphors sintered at 1300 °C for different durations. These phosphors exhibited diffraction peaks at 2*θ* values of 22.7°, 28.1°, 30.4°, 31.9°, 32.8°, 38.9°, 40.4°, 46.5°, 48.2°, 50.1°, 51.8°, 58.1°, 59.5°, and 60.8°. These characteristic peaks suggest that the aforementioned phosphors had a monoclinic structure (*a* ≠ *b* ≠ *c*, *α* = *β* = *γ* = 90°, P2_1_/a space group). In addition, the 2*θ* values of 24.9°, 31.1°, 35.4°, 43.9°, 45.1°, and 60.7° indicated the presence of the Sr_2_MgSi_2_O_7_ phase (JCPDS No. 75-1736) and α-Sr_2_SiO_4_ phase (JCPDS No. 39-1256). No Eu_2_O_3_ compound was found in the phosphors. As displayed in Figure 2, in the phosphors, each Si atom was surrounded by four oxygen atoms, which resulted in the formation of a four-coordination [SiO_4_] tetrahedral structure. Moreover, each Mg atom was surrounded by six oxygen atoms, which resulted in the formation of a [MgO_6_] octahedron. A Sr atom could occupy three available sites, which were located in different crystallographic environments.

The Sr(I), Sr(II), and Sr(III) sites exhibited ten-coordination, eight-coordination, and nine-coordination, respectively. Eu^2+^-doped [Sr_1−*x*_Eu*_x_*]_3_MgSi_2_O_8_ phosphors were obtained by reducing Eu^3+^ ions to Eu^2+^ ions in a reducing atmosphere during the sintering process. The ionic radius of Sr^2+^ is 1.01 Å, which is close to that of Eu^2+^ (1.12 Å). Mg^2+^ and Si^4+^ have smaller ionic radii (0.72 and 0.40 Å, respectively) than does Sr^2+^. Therefore, the diffraction peaks of Eu_2_O_3_ were not observed, which demonstrated that Eu^2+^ ions could be doped into the [Sr_0.99_Eu_0.01_]_3_MgSi_2_O_8_ lattice because of the similar ionic radii and valence of Sr^2+^ and Eu^2+^. As displayed in Figure 1, the intensity of the [Sr_0.99_Eu_0.01_]_3_MgSi_2_O_8_ signal increased as the sintering time increased from 1 to 10 h. Moreover, the intensities of the α-Sr_2_SiO_4_ and Sr_2_MgSi_2_O_7_ signals decreased with sintering time. Because the α-Sr_2_SiO_4_ and Sr_2_MgSi_2_O_7_ phases were formed within short sintering times or relatively low sintering temperatures, the [Sr_0.99_Eu_0.01_]_3_MgSi_2_O_8_ phosphors exhibited better crystalline structures at longer sintering times.

**Figure 3:**
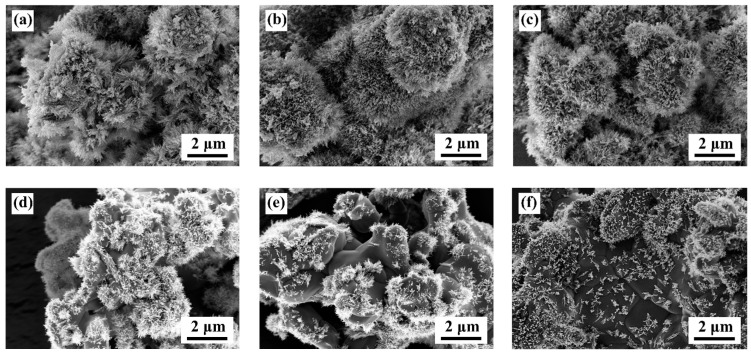
FE-SEM images of the [Sr_0.99_Eu_0.01_]_3_MgSi_2_O_8_ phosphors sintered for different durations: (**a**) 1, (**b**) 2, (**c**) 3, (**d**) 4, (**e**) 5, and (**f**) 6 h.

**Figure 4:**
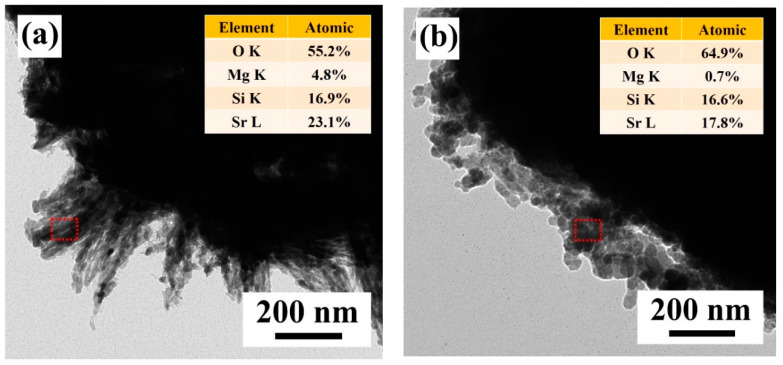
HR-TEM images of the [Sr_0.99_Eu_0.01_]_3_MgSi_2_O_8_ phosphors sintered for different durations: (**a**) 1 and (**b**) 5 h. The EDS results are shown in the inset.

Figure 3 displays the FE-SEM images of the [Sr_0.99_Eu_0.01_]_3_MgSi_2_O_8_ phosphors sintered at 1300 °C for different durations. When the sintering time was 1 h, the synthesized [Sr_0.99_Eu_0.01_]_3_MgSi_2_O_8_ phosphors exhibited a special surface morphology. The particles of these phosphors appeared similar to *Clavularia viridis*, which is a coral species, and exhibited many fine hairs on their surface. The number of fine hairs on the particle surface decreased as the sintering time increased from 1 to 6 h. In addition, to understand the microstructure of the fine hair, the prepared [Sr_0.99_Eu_0.01_]_3_MgSi_2_O_8_ phosphors were subjected to HR-TEM and energy dispersive X-ray spectroscopy (EDS) analyses (Figure 4). At a sintering time of 1 h, the atomic percentages of Sr, Mg, Si, and O in the fine hairs were 25.1%, 27.9%, 1.8%, and 45.2%, respectively. On the basis of this information and the XRD results (Figure 1), we infer that the Sr_2_SiO_4_ and Sr_2_MgSi_2_O_7_ phases were present in the fine hairs at a sintering time of 1 h. The element distribution images of the [Sr_0.99_Eu_0.01_]_3_MgSi_2_O_8_ phosphors are shown in Figure S1. The resulting presence of Sr, Si, and Mg can be found, and the element content was similar to the HR-TEM/EDS result (Figure 4). At a sintering time of 5 h, the fine hairs contained Sr, Mg, Si, and O, which indicates that the Sr_2_MgSi_2_O_7_ phase was present in the fine hairs at a sintering time of 5 h, almost the same as the detected atomic percentage and nominal compositions in quantity. The SEM images of the [Sr_0.99_Eu_0.01_]_3_MgSi_2_O_8_ phosphors sintered for different durations, whose BET specific surface area were 18.4 m^2^/g, 13.5 m^2^/g, 9.4 m^2^/g, 7.2 m^2^/g, 5.8 m^2^/g, and 2.5 m^2^/g, respectively, as shown in Figure 3a–f.

**Figure 5:**
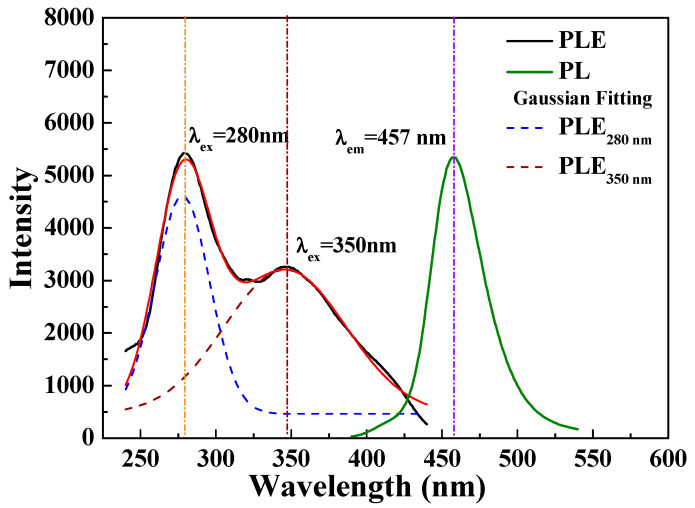
PLE and PL spectra of the [Sr_0.99_Eu_0.01_]_3_MgSi_2_O_8_ phosphors sintered for 5 h.

Figure 5 displays the PLE and PL spectra of the [Sr_0.99_Eu_0.01_]_3_MgSi_2_O_8_ phosphors sintered at 1300 °C for 5 h. The Eu^2+^ excitation band of the [Sr_0.99_Eu_0.01_]_3_MgSi_2_O_8_ phosphors can be fitted into two Gaussian components with peaks at 280 and 350 nm, which correspond to the 4f^7^(^8^S_7/2_)→4f^6^5d^1^(t_2g_) electron transition of Eu^2+^ [26]. Figure S2 shows the PLE spectra of the [Sr_0.99_Eu_0.01_]_3_MgSi_2_O_8_ phosphors sintered for different durations. These spectra exhibit two broad bands ranging from 240 to 320 nm and from 330 to 410 nm, with peaks at 280 and 350 nm, which are assigned to the transitions between the ground state 4f^7^ and the crystal-field split state 4f^6^5d^1^. As the sintering time increased, the excitation intensity increased and reached a maximum value at a sintering time of 5 h. The aforementioned results demonstrate that as the sintering duration increased from 1 to 5 h, the crystallinity (Figure 1), particle morphologies and sizes (Figure 3), and PLE intensities of the phosphors increased.

**Figure 6:**
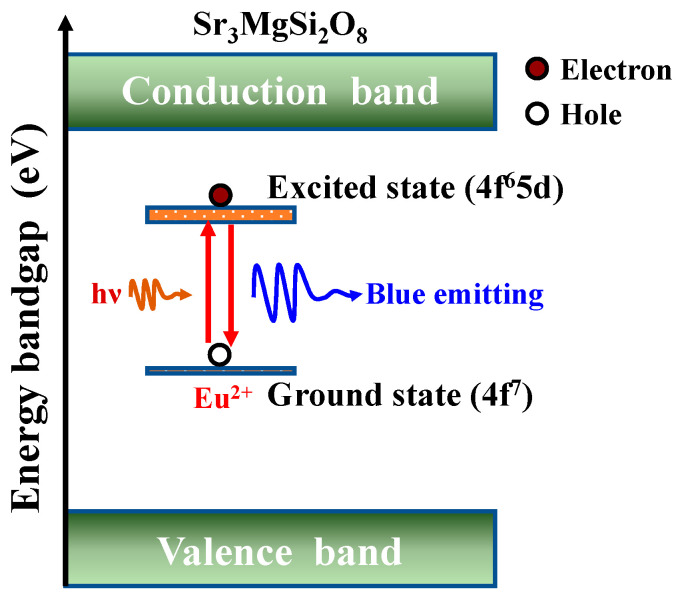
The electronic mechanism of the [Sr_0.99_Eu_0.01_]_3_MgSi_2_O_8_ phosphors.

Figure S3 shows the PL spectra of the [Sr_0.99_Eu_0.01_]_3_MgSi_2_O_8_ phosphors sintered at 1300 °C for different durations. The emission spectra corresponding to 280 nm excitation contain a single band at around 457 nm. As displayed in Figure S2, the [Sr_0.99_Eu_0.01_]_3_MgSi_2_O_8_ phosphors exhibited the highest emission peak intensities when the sintering duration was 5 h, the Sr_3_MgSi_2_O_8_ has a space group of P21/a, and the unit cell contains three Sr sites: one 12-coordinated Sr(I) site and two 10-coordinated Sr(II, III) sites [27]. The broad band at around 457 nm is attributed to the 4f^6^5d–4f^7^ transition at the Sr^2+^(I) site, where Sr^2+^ is substituted by Eu^2+^ [28,29]. The electronic mechanism of the [Sr_0.99_Eu_0.01_]_3_MgSi_2_O_8_ phosphors is shown in Figure 6. The 4f^6^5d–4f^7^ transition belongs to the electronic dipole-allowed transition, based on the Laporte selection rule. Kim et al. indicated that the 570 nm band to Eu^2+^ ions at the Sr^2+^ (II, III) sites occurs at high Eu^2+^ doping concentrations in Sr_3_MgSi_2_O_8_ [28]. Figure S2 does not indicate an emission peak at 570 nm; thus, only Eu^2+^ ions substituted Sr^2+^ at the Sr^2+^(I) site. The full width at half maximum (FWHM) of the broad band of emission peaks were approximately 50, 46, 43, 41, and 40 nm as the sintered for 1 to 5 h. This result was caused by the electron on the outer 5*d*-orbital of the atom, while the emission peak of the [Sr_0.99_Eu_0.01_]_3_MgSi_2_O_8_ phosphors was easily influenced by the external environment.

**Figure 7:**
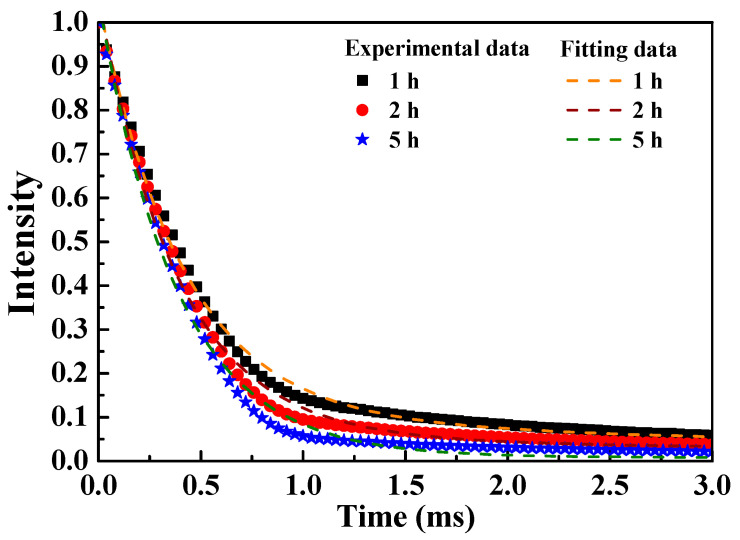
Decay times of the [Sr_0.99_Eu_0.01_]_3_MgSi_2_O_8_ phosphors sintered for different durations.

Figure 7 displays the fluorescent decay curves of the [Sr_0.99_Eu_0.01_]_3_MgSi_2_O_8_ phosphors excited at 280 nm and monitored at 457 nm. These data fit well with a double-exponential curve. The aforementioned curves indicate the possible interactions between Eu^2^^+^ ions and suggest that these ions occupied the cationic sites (Sr^2+^). To calculate the luminescence lifetimes, all the fluorescent decay curves were fitted using the double-exponential equation of Sahu et al. [30], which is expressed as follows:

*I* = *A*_1_exp(−*t*/*τ*_1_) + *A*_2_exp(−*t*/*τ*_2_) (1)

where *I* is the PL intensity, *A*_1_ and *A*_2_ are the fitting parameters, and *τ*_1_ and *τ*_2_ are the decay constants of the exponential components.

On the basis of the aforementioned equation, the average luminescence lifetimes (τ*) of a rare-earth ion can be calculated using the following equation [31]:

τ* = (*A*_1_*τ*_1_^2^ _+_ *A*_2_*τ*_2_^2^)/(*A*_1_*τ*_1 +_ *A*_2_*τ*_2_) (2)

The average luminescence lifetimes of the [Sr_0.99_Eu_0.01_]_3_MgSi_2_O_8_ phosphors were calculated to be 3.406, 3.191, and 1.143 ms for the sintering durations of 1, 2, and 5 h, respectively. The parameter τ* decreased with sintering time. This phenomenon might be attributed to the energy transfer between the Eu^2^^+^ ions located at the Sr^2^^+^ sites [32].

**Figure 8:**
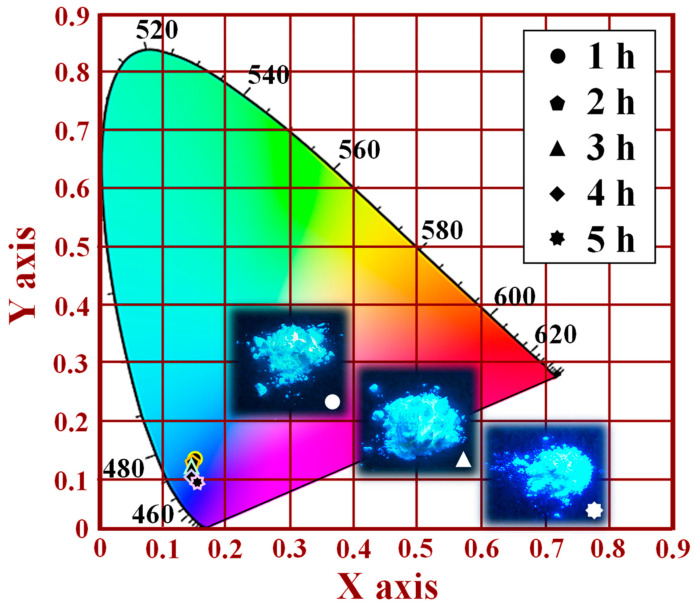
CIE chromaticity diagram of the [Sr_0.99_Eu_0.01_]_3_MgSi_2_O_8_ phosphors sintered for different durations. Images of the phosphors are shown in the inset.

Figure 8 shows the CIE chromaticity results of the [Sr_0.99_Eu_0.01_]_3_MgSi_2_O_8_ phosphors as a function of the sintering duration. The CIE (1931 chromaticity) diagram can be used to describe the color purity of the luminescent emissions of phosphors. In this study, a CIE chromaticity diagram was obtained for an excitation wavelength of 280 nm. The color coordinates of the [Sr_0.99_Eu_0.01_]_3_MgSi_2_O_8_ phosphors sintered for 1, 2, 3, 4, and 5 h were (0.1659, 0.1382), (0.1612, 0.1256), (0.1593, 0.1211), (0.1549, 0.1111), and (0.1527, 0.1006), as displayed in Figure 8. The CIE chromaticity diagram indicates that as the sintering duration increased from 1 to 5 h, the emissions of the [Sr_0.99_Eu_0.01_]_3_MgSi_2_O_8_ phosphors changed from being light blue to navy blue. Thus, a sintering temperature of 1300 °C and a sintering duration of 5 h are optimal settings for the synthesis of a blue phosphor. The aforementioned results indicate that sintering duration is the main factor affecting the crystalline structure and PL properties of [Sr_0.99_Eu_0.01_]_3_MgSi_2_O_8_ phosphors.

Images of the [Sr_0.99_Eu_0.01_]_3_MgSi_2_O_8_ phosphors sintered for different durations under ultraviolet (UV) light irradiation are shown in the inset of Figure 8 and in Figure S4. The brightness of the [Sr_0.99_Eu_0.01_]_3_MgSi_2_O_8_ phosphors increased with sintering duration. The phosphors sintered at 1300 °C for 5 h were very bright.

**Figure 9:**
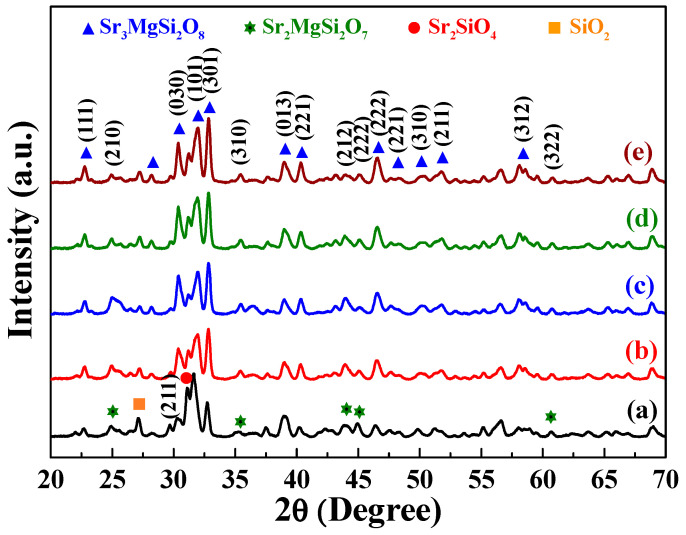
XRD patterns of the [Sr_0.99_Eu_0.01_]_3_MgSi_2_O_8_ phosphors sintered at different temperatures: (a) 1200, (b) 1250, (c) 1300, (d) 1350, and (e) 1400 °C.

Sintering temperature affects the PL properties and structure of phosphors. Therefore, we attempted to determine the optimal sintering temperature for preparing [Sr_0.99_Eu_0.01_]_3_MgSi_2_O_8_ phosphors. XRD patterns of the [Sr_0.99_Eu_0.01_]_3_MgSi_2_O_8_ phosphors sintered using the solid-state method at temperatures of 1200, 1250, 1300, 1350 and 1400 °C for 5 h are depicted in Figure 9. Figure 9a shows the diffraction peaks of the phosphor sintered at 1200 °C. This phosphor exhibited main diffraction peaks at 2*θ* values of 22.7°, 28.1°, 30.4°, 31.9°, 32.8°, 38.9°, 40.4°, 46.5°, 48.2°, 50.1°, 51.8°, 58.1°, 59.5°, and 60.8°. This set of XRD peaks is similar to that observed for Sr_3_MgSi_2_O_8_ (JCPDS No. 10-0075). In addition, the [Sr_0.99_Eu_0.01_]_3_MgSi_2_O_8_ phosphor contained the Sr_2_MgSi_2_O_7_ (JCPDS No. 75-1736) and α-Sr_2_SiO_4_ (JCPDS No. 39-1256). The intensity of the [Sr_0.99_Eu_0.01_]_3_MgSi_2_O_8_ signal increased with sintering temperature from 1200 to 1400 °C. Moreover, the intensities of the Sr_2_MgSi_2_O_7_ and α-Sr_2_SiO_4_ signals decreased with sintering temperature.

The aforementioned results indicate that the row material of SrCO_3_ decomposed into SrO and CO_2_, then SrO reacted with SiO_2_ to form Sr_2_SiO_4_, and finally SrO and MgO reacted with SiO_2_ to form the Sr_2_MgSi_2_O_7_ and Sr_3_MgSi_2_O_8_ phases. When the sintering temperature was lower than 1000 °C, the following reaction occurred:

SrCO_3_ → SrO + CO_2_ (3)

When the sintering temperature was between 1000 and 1200 °C, the following reaction occurred [33,34]:

2SrO+SiO_2_ → Sr_2_SiO_4_ (4)

When the sintering temperature was between 1200 and 1300 °C, the following reaction occurred [35]:

2SrO + MgO + 2SiO_2_ → Sr_2_MgSi_2_O_7_ (5)

At 1450 °C, the [Sr_0.99_Eu_0.01_]_3_MgSi_2_O_8_ phosphor melted. Consequently, the crystalline structures and PL properties of the [Sr_0.99_Eu_0.01_]_3_MgSi_2_O_8_ phosphors were not examined at sintering temperatures higher than 1450 °C.

**Figure 10:**
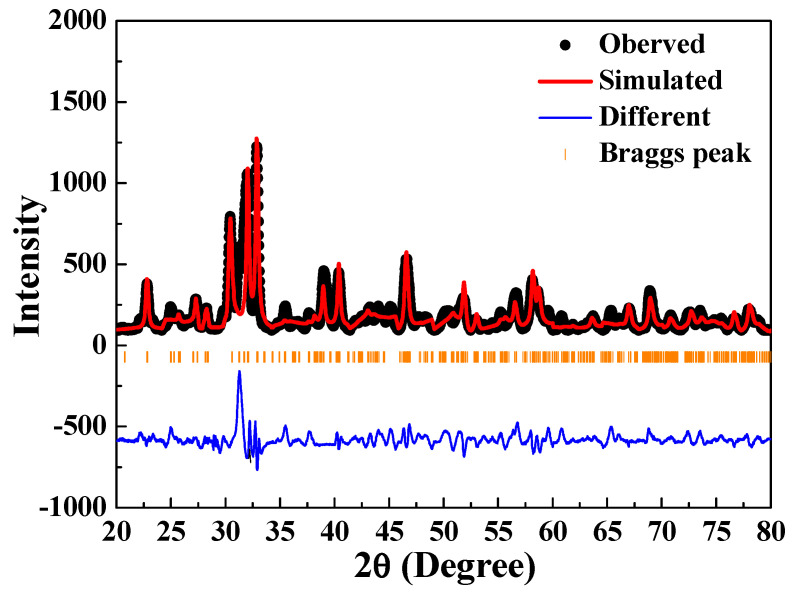
Results of the Rietveld refinement performed for the [Sr_0.99_Eu_0.01_]_3_MgSi_2_O_8_ phosphor sintered at 1400 °C.

The findings for the crystal structure of the [Sr_0.99_Eu_0.01_]_3_MgSi_2_O_8_ phosphor sintered at 1400 °C was fitted using the following parameters: *a* = 5.341 Å, *b* = 9.700 Å, and *c* = 7.184 Å (Sr_3_MgSi_2_O_8_ phosphors). Subsequently, Rietveld refinement was conducted on the XRD data of this phosphor (Figure 10). The final refinement convergence was achieved when *χ*^2^ = 5.42, which is marginally higher than the optimal value *χ*^2^ value of <2. This result was due to the coexistence of the Sr_2_MgSi_2_O_7_ (2*θ* = 29.7° and 30.2°) and α-Sr_2_SiO_4_ (2*θ* = 35.4°, 43.9°, 45.1°, and 60.7°) phases in the aforementioned phosphor. The remaining diffraction peak of 2*θ* values, in addition to those mentioned above, were assigned to the [Sr_0.99_Eu_0.01_]_3_MgSi_2_O_8_ phase. It demonstrated that the Sr^2+^ ions were substituted by Eu^2+^ ions in the [Sr_0.99_Eu_0.01_]_3_MgSi_2_O_8_ phosphors.

**Figure 11:**
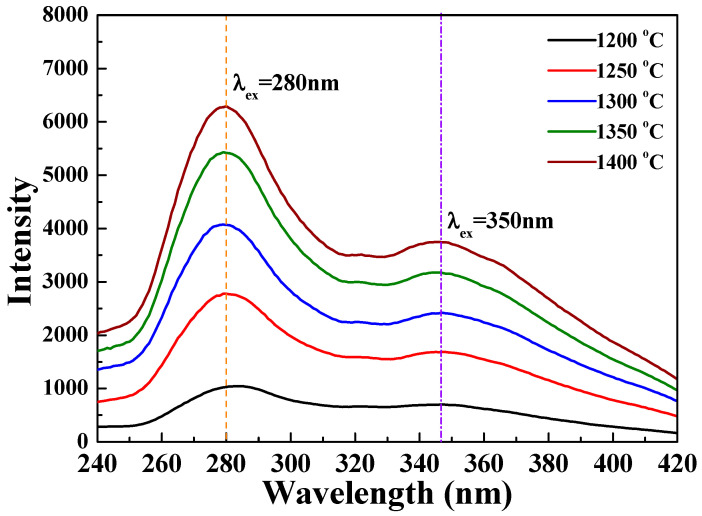
PLE spectra of the [Sr_0.99_Eu_0.01_]_3_MgSi_2_O_8_ phosphors sintered at different temperatures.

**Figure 12:**
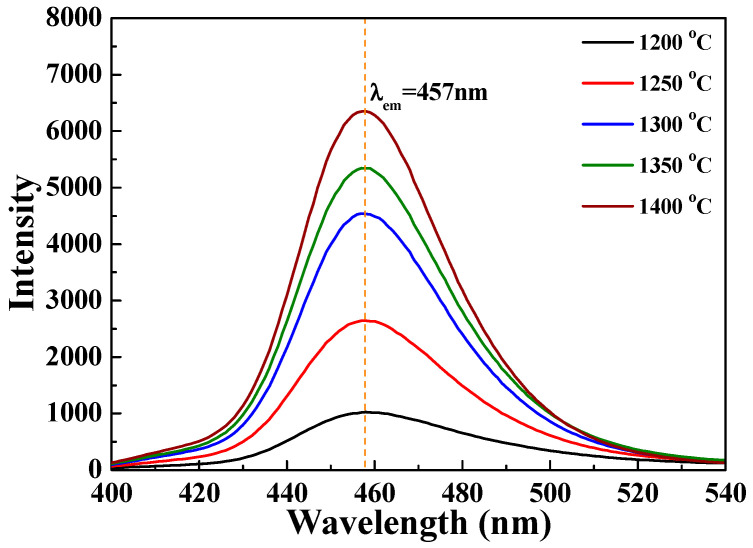
PL spectra of the [Sr_0.99_Eu_0.01_]_3_MgSi_2_O_8_ phosphors sintered at different temperatures.

Figure 11 and Figure 12 depict the PLE and PL spectra of the [Sr_0.99_Eu_0.01_]_3_MgSi_2_O_8_ phosphors sintered at different temperatures. As the sintering temperature increased, the PLE intensity also increased, and the maximum PLE intensity was achieved when the sintering temperature was 1400 °C (Figure 11). As depicted in Figure 12, the PL intensity of the [Sr_0.99_Eu_0.01_]_3_MgSi_2_O_8_ phosphors increased with sintering temperature. The [Sr_0.99_Eu_0.01_]_3_MgSi_2_O_8_ phosphor sintered at 1400 °C exhibited the highest PL intensity, and the broad and asymmetric band with an FWHM value of 38 nm was observed at around 457 nm. The FWHM of the broad band of emission peaks were approximately 38, 40, 43 and 45 nm as the sintered temperature decreased from 1400 °C to 1200 °C. The blue emission band of the [Sr_0.99_Eu_0.01_]_3_MgSi_2_O_8_ phosphors at 457 nm was attributed to the 5*d*–4*f* electron transition of Eu^2+^.

**Figure 13:**
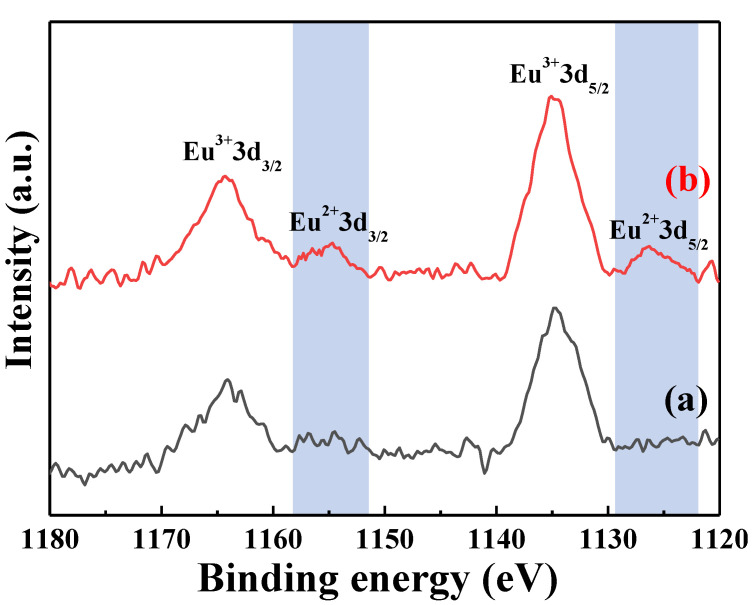
XPS spectra of the [Sr_0.99_Eu_0.01_]_3_MgSi_2_O_8_ phosphors sintered at different temperatures. (a) 900 °C and (b) 1400 °C.

**Figure 14:**
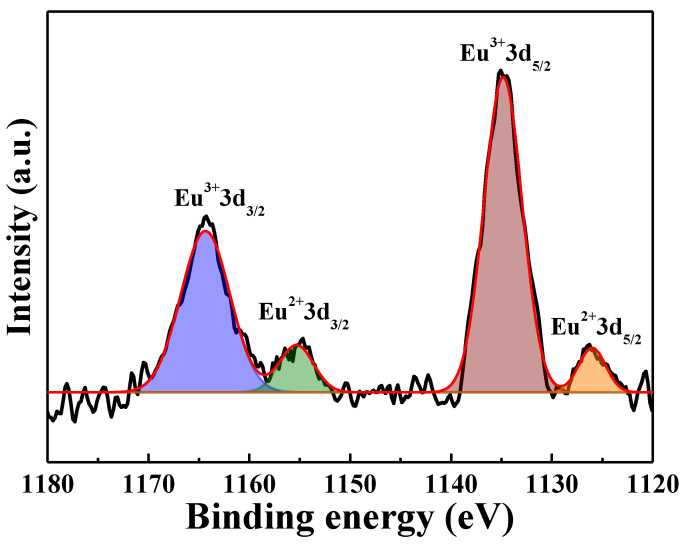
XPS spectra of the [Sr_0.99_Eu_0.01_]_3_MgSi_2_O_8_ phosphors sintered at 1400 °C.

Figure 13 shows the Eu 3d XPS spectra of the [Sr_0.99_Eu_0.01_]_3_MgSi_2_O_8_ phosphors sintered at different temperatures. The results shows that there is no Eu^2+^-related peaks at the sintered temperature of 900 °C (Figure 13a), and the Eu^2+^ peak appeared at the sintering temperature of 1400 °C (Figure 13b). The Eu 3d XPS spectra of the [Sr_0.99_Eu_0.01_]_3_MgSi_2_O_8_ phosphors sintered at 1400 °C is shown in Figure 14, revealing the Eu 3d peak deconvolution of the electron binding energies of Eu^3+^ 3d_3/2_ (1164 eV), Eu^2+^ 3d_3/2_ (1155 eV), Eu^3+^ 3d_5/2_ (1134 eV), and Eu^2+^ 3d_5/2_ (1125 eV). This result demonstrated that the Eu^3+^ ions are successfully reduced to Eu^2+^ ions at a 1400 °C sintering temperature. In general, Eu^3+^→Eu^2+^ reduction requires a higher temperature in the reducing atmosphere.

Figure S5 displays the fluorescent decay curves of the [Sr_0.99_Eu_0.01_]_3_MgSi_2_O_8_ phosphors excited at 280 nm and monitored at 457 nm. The data fit well with a double-exponential curve. The average luminescence lifetimes of the [Sr_0.99_Eu_0.01_]_3_MgSi_2_O_8_ phosphors sintered at 1200, 1300, and 1400 °C were calculated from Equation (2) to be 1.074, 1.144, and 1.197 ms, respectively. The parameter τ* decreased with sintering temperature. This result demonstrates that energy transfer occurred between the Eu^2^^+^ ions located at the Sr^2^^+^ sites [32].

**Figure 15:**
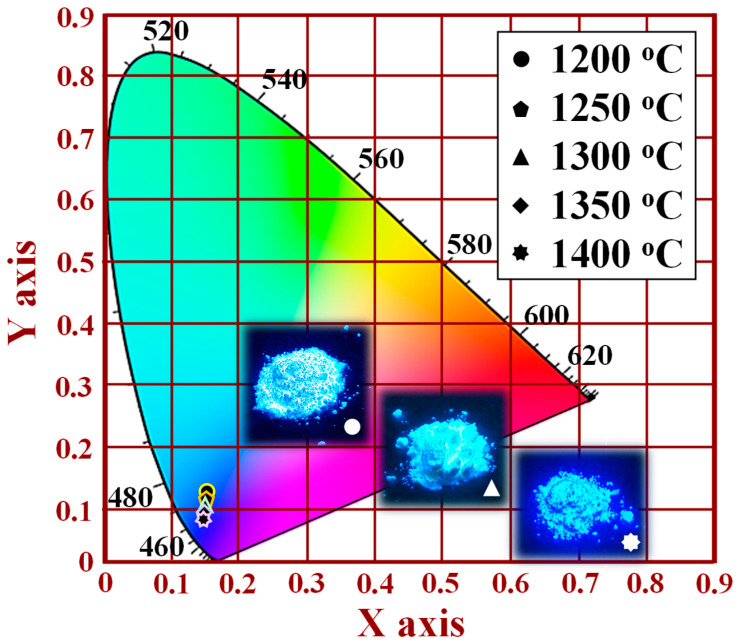
CIE chromaticity diagram for the [Sr_0.99_Eu_0.01_]_3_MgSi_2_O_8_ phosphors sintered at different temperatures.

Figure 15 shows the CIE chromaticity coordinates and photographs of the [Sr_0.99_Eu_0.01_]_3_MgSi_2_O_8_ phosphors sintered at different temperatures. The CIE chromaticity diagram was obtained for an excitation wavelength of 280 nm. When the sintering temperature was increased from 1200 to 1400 °C, the CIE chromaticity coordinates shifted from a light blue region (*x* = 0.1659, *y* = 0.1382) to an ultramarine blue region (*x* = 0.1494, *y* = 0.0942). Therefore, the optimal sintering temperature in the production of blue phosphors is 1400 °C. Images of the [Sr_0.99_Eu_0.01_]_3_MgSi_2_O_8_ phosphors sintered at different temperatures under UV light irradiation are displayed in the inset of Figure 15 and in Figure S6. The brightness of the [Sr_0.99_Eu_0.01_]_3_MgSi_2_O_8_ phosphors increased with sintering temperature. The highest brightness occurred at a sintering temperature of 1400 °C.

## 4. Conclusions

In this study, Eu^2+^-doped [Sr_1−x_Eu_x_]_3_MgSi_2_O_8_ phosphors were prepared in a reducing atmosphere by using a solid-state reaction method, and the photoluminescence properties of these phosphors were investigated. The optimal sintering temperature and duration for the preparation of the [Sr_0.99_Eu_0.01_]_3_MgSi_2_O_8_ phosphors was found to be 1400 °C and 5 h, respectively. The blue emission of these phosphors at 457 nm is attributed to the 5*d*–4*f* electron transition of Eu^2+^. In addition, the average decay time of the [Sr_0.99_Eu_0.01_]_3_MgSi_2_O_8_ phosphors sintered at 1400 °C for 5 h was calculated to be 1.197 ms. The CIE chromaticity coordinates of the phosphors sintered at 1400 °C were (*x* = 0.1494, *y* = 0.0942), and this point lies in an ultramarine blue region in the CIE chromaticity diagram. [Sr_0.99_Eu_0.01_]_3_MgSi_2_O_8_ is promising as a blue phosphor in RGB-W-LEDs.

## Data Availability

Not applicable.

## Supplementary Materials

The following supporting information can be downloaded at: https://www.mdpi.com/article/10.3390/nano12152706/s1, Figure S1: SEM/EDS mapping images of the [Sr_0.99_Eu_0.01_]_3_MgSi_2_O_8_ phosphor sintered at 1300 °C for 1 h. (a) SEM images, (b) Sr element, (c) Si element and (d) Mg element. Figure S2: PLE patterns of the [Sr_0.99_Eu_0.01_]_3_MgSi_2_O_8_ phosphors sintered at different times; Figure S3: PL patterns of the [Sr_0.99_Eu_0.01_]_3_MgSi_2_O_8_ phosphors sintered at different times; Figure S4: The photo-images of the [Sr_0.99_Eu_0.01_]_3_MgSi_2_O_8_ phosphors sintered at different times under UV light irradiation. (a) 1 h, (b) 2 h, (c) 3 h, (d) 4 h and (e) 5 h, respectively; Figure S5: Decay times of [Sr_0.99_Eu_0.01_]_3_MgSi_2_O_8_ phosphors sintered at different temperatures; Figure S6: The photo-images of the [Sr_0.99_Eu_0.01_]_3_MgSi_2_O_8_ phosphors sintered at different temperatures under UV light irradiation. (a) 1200 °C, (b) 1250 °C, (c) 1300 °C, (d) 1350 °C and (e) 1400 °C, respectively.
