# Supplementary material for: Synthesis and Luminescence Properties of Eu2+-Doped Sr3MgSi2O8 Blue Light-Emitting Phosphor for Application in Near-Ultraviolet Excitable White Light-Emitting Diodes
Source: Nanomaterials (Basel). 2022 Aug 6;12(15):2706. doi: 10.3390/nano12152706 (PMC9370580; doi:10.3390/nano12152706)
Supplement: Supplementary file 1 [file nanomaterials-12-02706-s001.zip › nanomaterials-1786720-supplementary.pdf]

nanomaterials

Supplementary Materials

Synthesis and Luminescence Properties of Eu^{2+}-Doped $Sr_3MgSi_2O_8$ Blue Light-Emitting Phosphor for Application in Near-Ultraviolet Excitable White Light-Emitting Diodes

Chou-Yuan Lee [1], Chia-Ching Wu [2,*], Hsin-Hua Li [3] and Cheng-Fu Yang [3,4,*]

[1] School of Big Data, Fuzhou University of International Studies and Trade, Fuzhou 350202, China; lqy@fzfu.edu.cn
[2] Department of Applied Science, National Taitung University, Taitung 95092, Taiwan
[3] Department of Chemical and Materials Engineering, National University of Kaohsiung, Kaohsiung 811726, Taiwan; hsinli1994@gmail.com
[4] Department of Aeronautical Engineering, Chaoyang University of Technology, Taichung 413310, Taiwan
* Correspondence: ccwu@nttu.edu.tw (C.-C.W.); cfyang@nuk.edu.tw (C.-F.Y.)

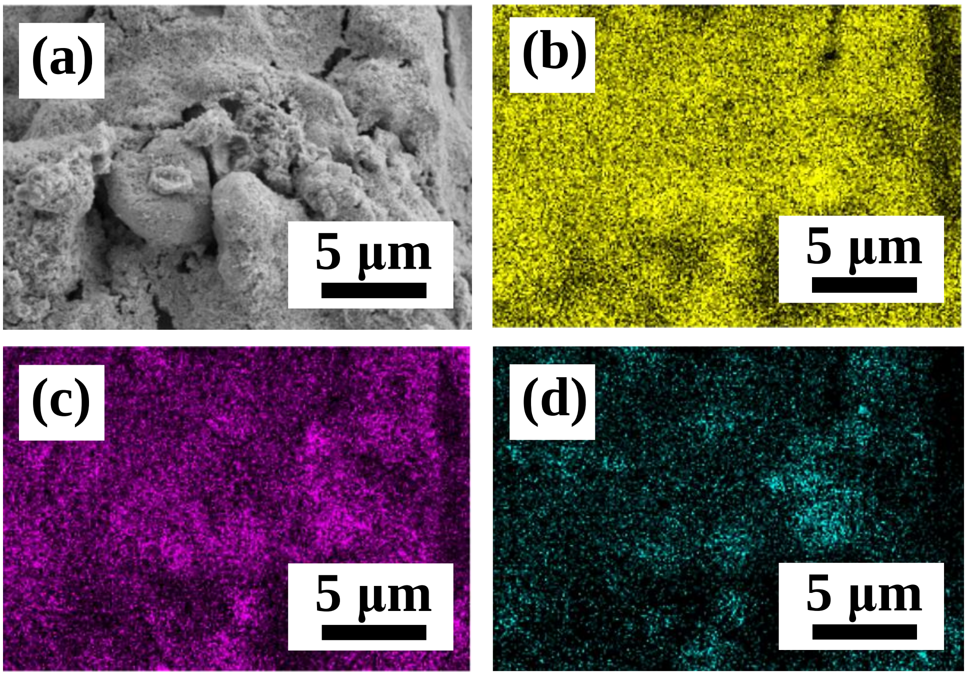

Figure S1. SEM/EDS mapping images of the $[Sr_{0.99}Eu_{0.01}]3MgSi_2O_8$ phosphor sintered at 1300 °C for 1 h. (**a**) SEM images, (**b**) Sr element, (**c**) Si element and (**d**) Mg element.

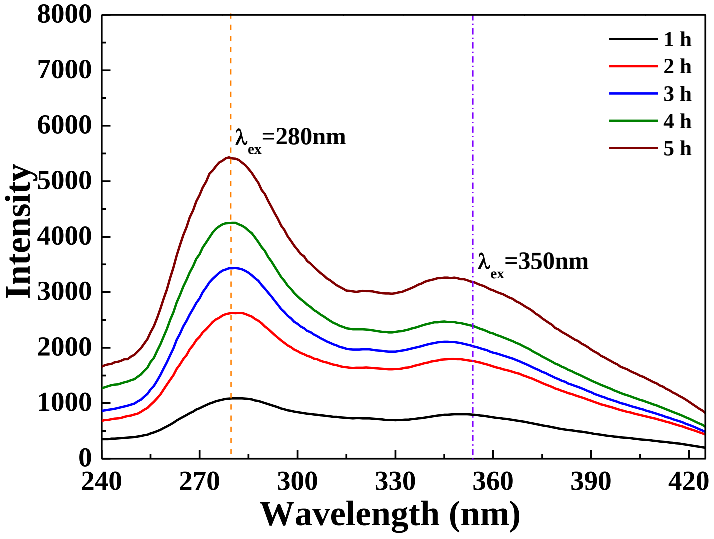

Figure S2. PLE patterns of the $[Sr_{0.99}Eu_{0.01}]3MgSi_2O_8$ phosphors sintered at different times.

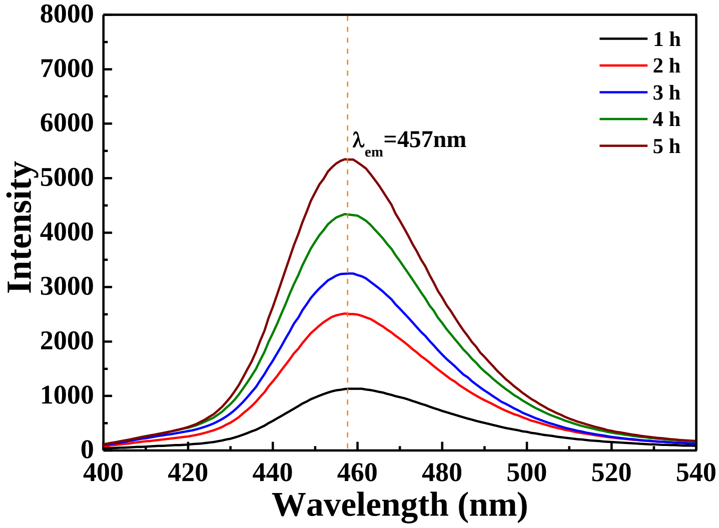

Figure S3. PL patterns of the $[Sr_{0.99}Eu_{0.01}]3MgSi_2O_8$ phosphors sintered at different times.

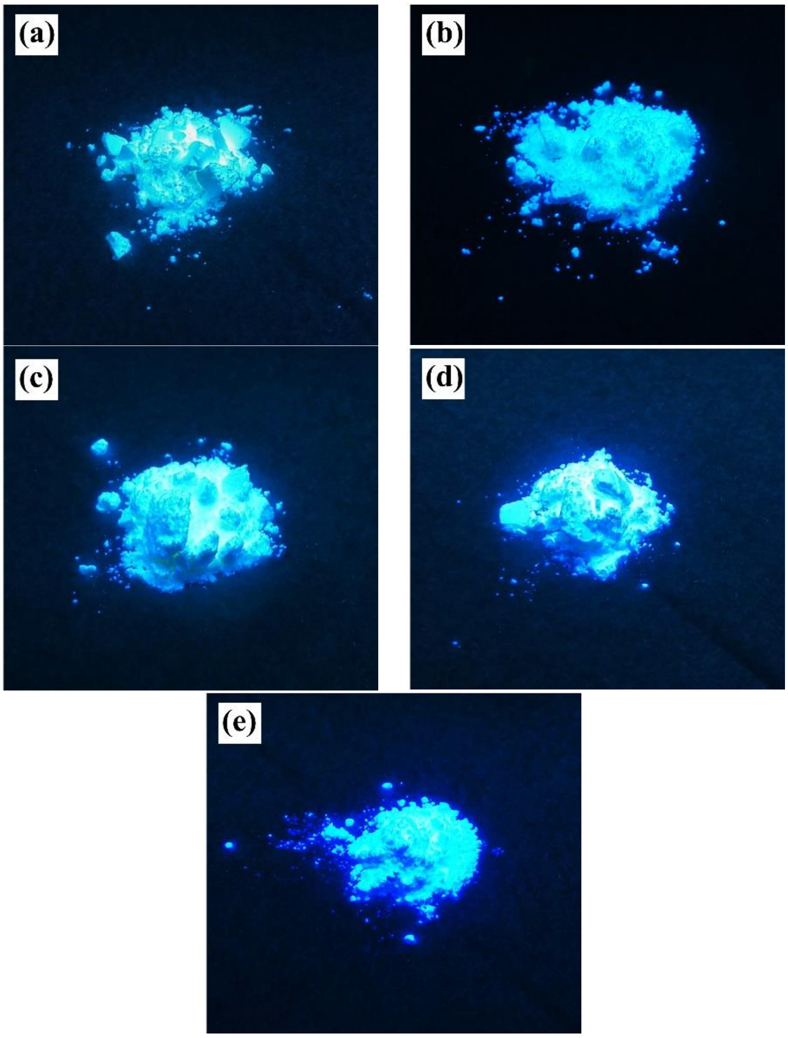

Figure S4. The photo-images of the $[Sr_{0.99}Eu_{0.01}]_3MgSi_2O_8$ phosphors sintered at different times under UV light irradiation. (**a**) 1 h, (**b**) 2 h, (**c**) 3 h, (**d**) 4 h and (**e**) 5 h, respectively.

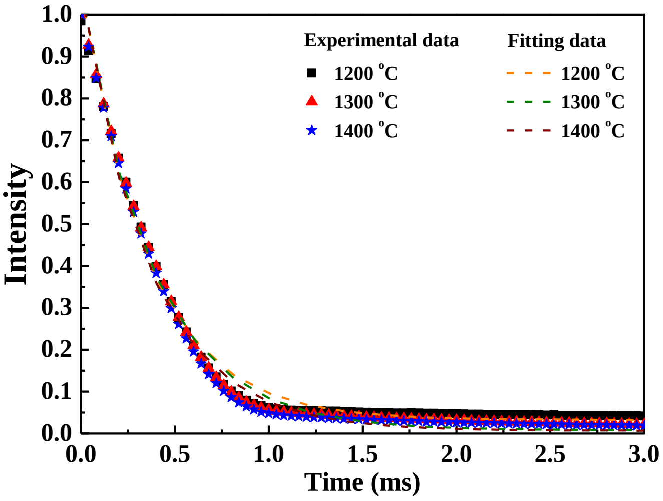

Figure S5. Decay times of $[Sr_{0.99}Eu_{0.01}]_3MgSi_2O_8$ phosphors sintered at different temperatures.

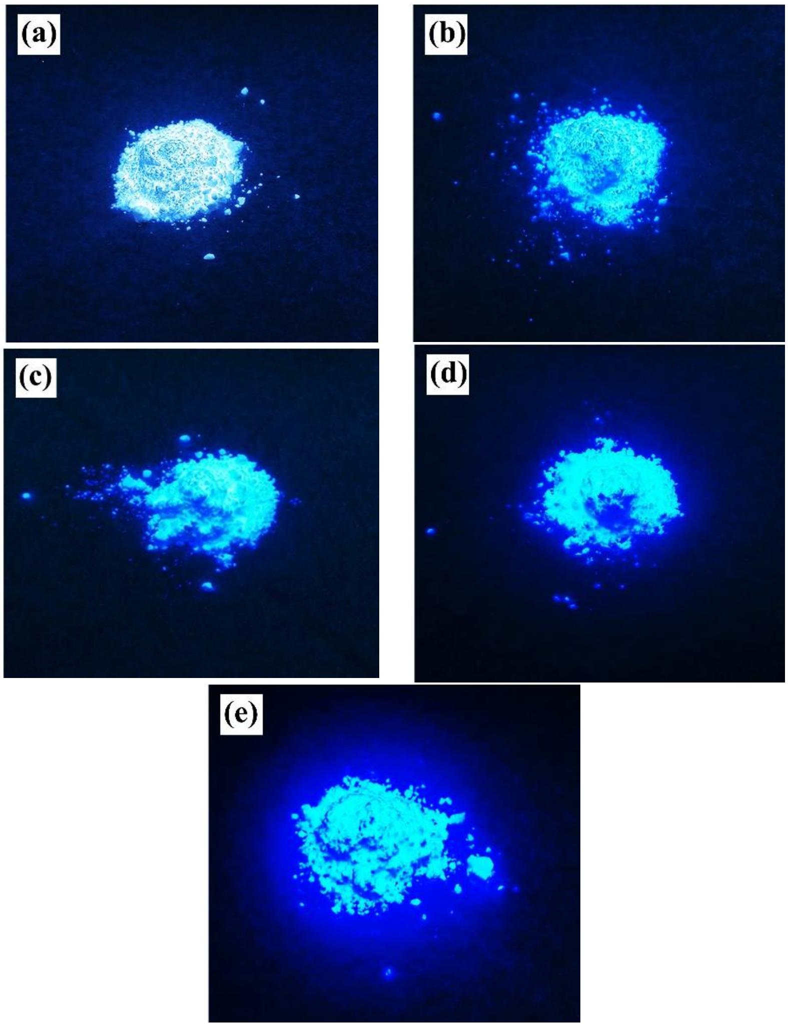

Figure S6. The photo-images of the $[Sr_{0.99}Eu_{0.01}]_3MgSi_2O_8$ phosphors sintered at different temperatures under UV light irradiation. (**a**) 1200 °C, (**b**) 1250 °C, (**c**) 1300 °C, (**d**) 1350 °C and (**e**) 1400 °C, respectively.
